# Supplementary material for: Solvothermal In-Situ Synthesis of MIL-53(Fe)@Carbon Felt Photocatalytic Membrane for Rhodamine B Degradation
Source: Int J Environ Res Public Health. 2023 Mar 4;20(5):4571. doi: 10.3390/ijerph20054571 (PMC10001776; doi:10.3390/ijerph20054571)
Supplement: Supplementary file 1 [file ijerph-20-04571-s001.zip › ijerph-2217697-supplementary.pdf]

International Journal of
Environmental Research and Public Health

MDPI

Supporting Information

Solvothermal In-Situ Synthesis of MIL-53(Fe)@Carbon Felt Photocatalytic Membrane for Rhodamine B Degradation

Shuyan Yu [1,2,3], Huiying Zhang [1,2,3] and Congju Li [1,2,3,*]

1 School of Energy and Environmental Engineering, University of Science and Technology Beijing, Beijing 100083, China
2 Beijing Key Laboratory of Resource-Oriented Treatment of Industrial Pollutants, Beijing 100083, China
3 Energy Conservation and Environmental Protection Engineering Research Center in Universities of Beijing, Beijing 100083, China
* Correspondence: congjuli@126.com

Supporting Information: 7 figures.

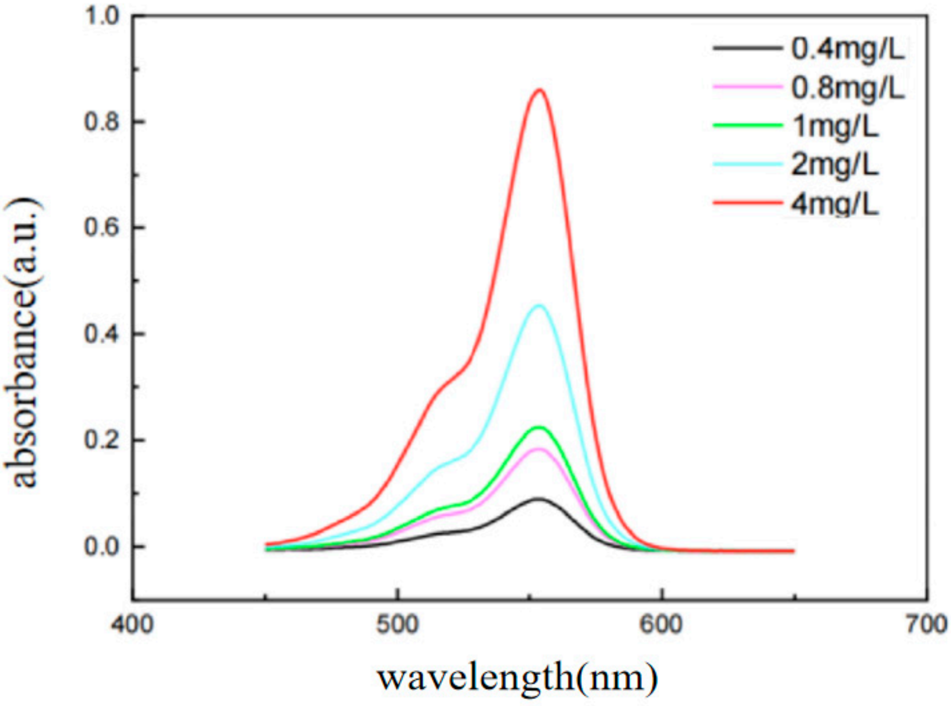

Figure S1. UV-Vis absorption spectrum of RhB standard solution.

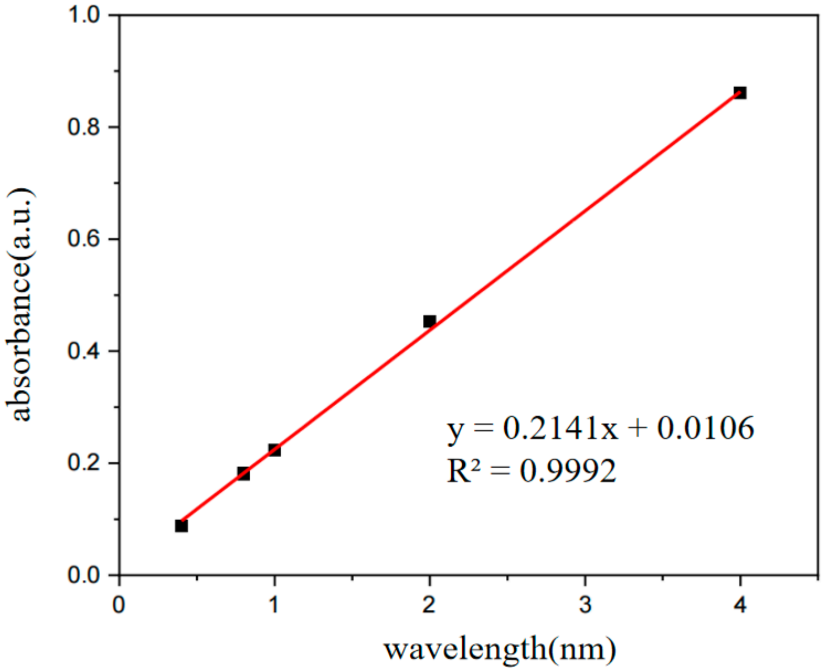

Figure S2. Gradient concentration RhB UV-visible absorption standard curve (Reactor: Cylindrical Pyrex container, Current:15 A, test in 100 ml of 1 mg/L RhB in water with 0.15 g of MIL-53(Fe)@CF photocatalytic membrane).

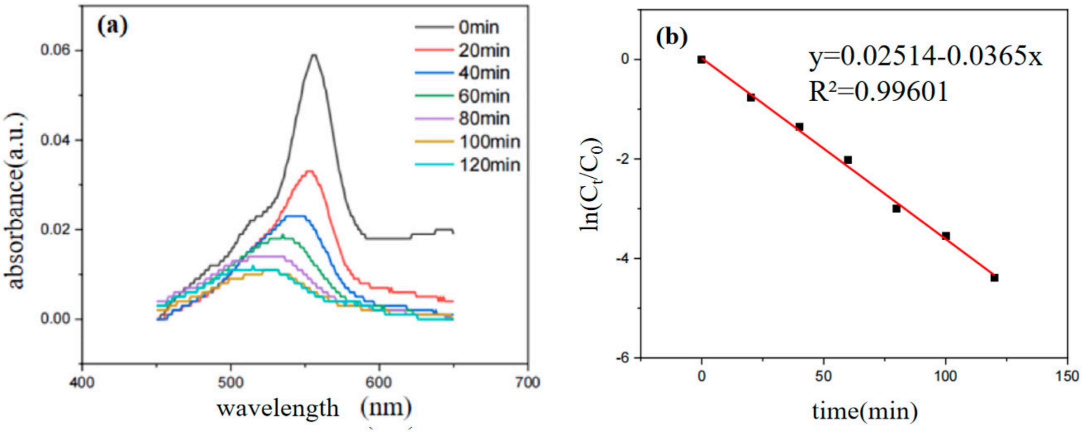

Figure S3. UV-Vis absorption spectrum (a) and kinetic curve (b) of the electron catcher H_2O_2 for photocatalytic oxidation of RhB (Reactor: pH at 4.5, MIL-53(Fe)@CF photocatalytic membrane 150 mg, RhB 1 mg/L, H_2O_2 1 mmol/L).

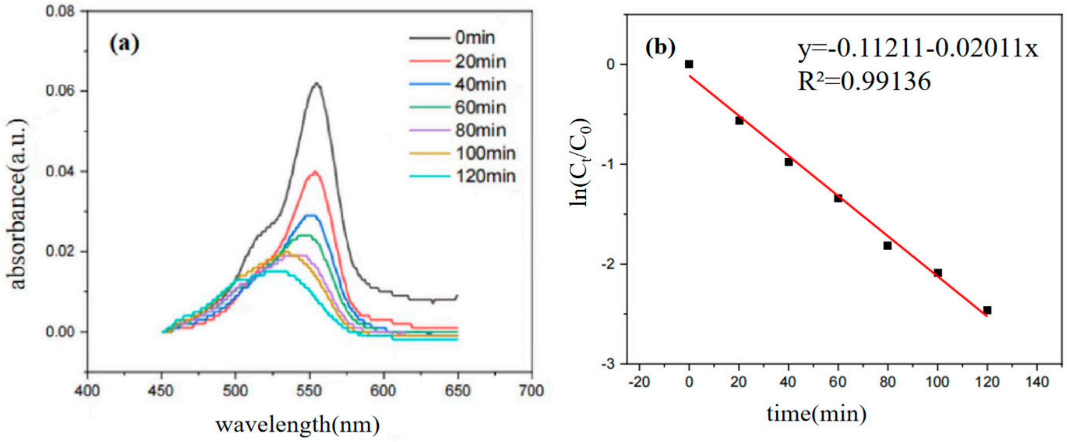

Figure S4. UV-Vis absorption spectrum (a) and kinetic curve (b) of electron trap $(NH_4)_2S_2O_8$ for photocatalytic oxidation of RhB (Reactor: pH at 4.5, MIL-53(Fe)@CF photocatalytic membrane 150 mg, RhB 1 mg/L, $(NH_4)_2S_2O_8$ 1 mmol/L).

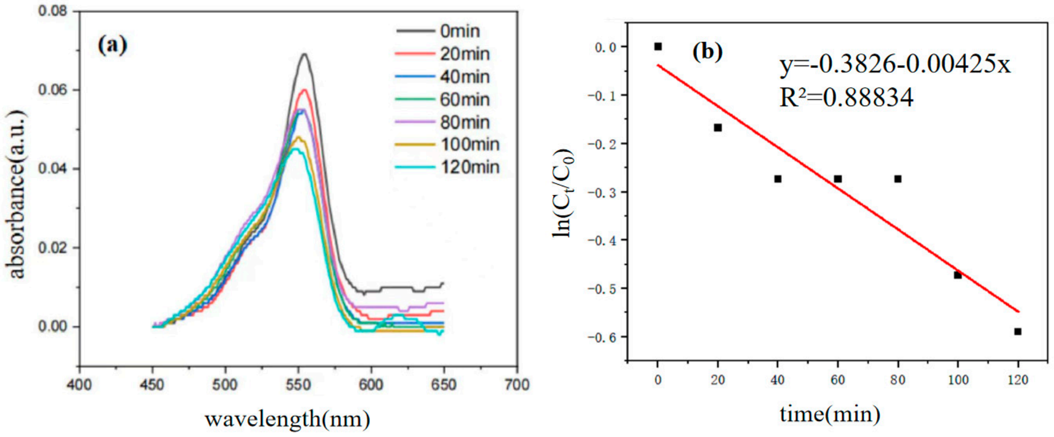

Figure S5. UV-Vis absorption spectrum (a) and kinetic curve (b) of electron trap $KBrO_3$ for photocatalytic oxidation of RhB (Reactor: pH at 4.5, MIL-53(Fe)@CF photocatalytic membrane 150 mg, RhB 1 mg/L, $KBrO_3$ 1 mmol/L).

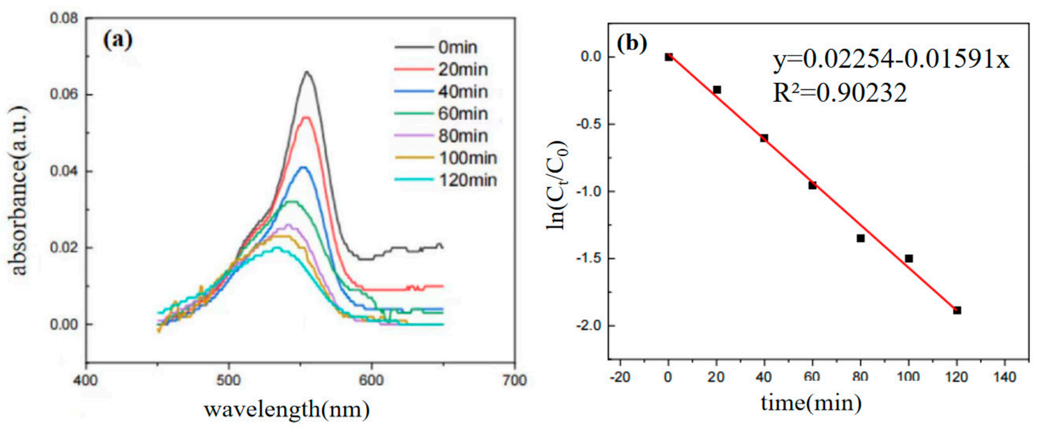

Figure S6. UV-Vis absorption spectrum (a) and kinetic curve (b) of photocatalytic oxidation of RhB with pH at 3.5. (Reactor: pH at 3.5, MIL-53(Fe)@CF photocatalytic membrane 150 mg, RhB 1 mg/L, H_2O_2 1 mmol/L).

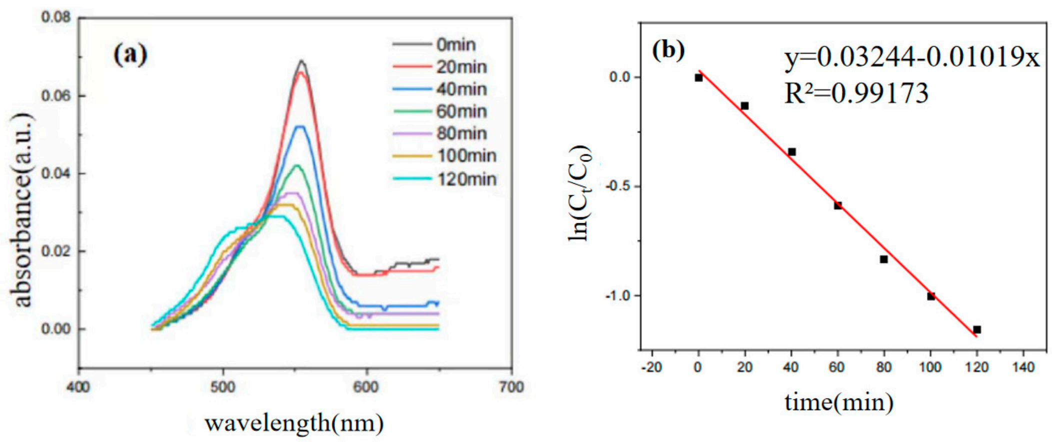

Figure S7. UV-Vis absorption spectrum (a) and kinetic curve (b) of photocatalytic oxidation of RhB with pH at 5.5. (Reactor: pH at 5.5, MIL-53(Fe)@CF photocatalytic membrane 150 mg, RhB 1mg/L, H_2O_2 1mmol/L)
